# Impact of the armed conflict on victims and support workers' mental health in Soacha, Colombia

**DOI:** 10.7705/biomedica.7250

**Published:** 2025-03-28

**Authors:** Sandra Elizabeth Piñeros, Nathaly Garzón, Zulma Consuelo Urrego, Nikki Coghill, Daniel Samacá, Javier Hernando Eslava

**Affiliations:** 1 Departamento de Psiquiatría, Facultad de Medicina, Universidad Nacional de Colombia, Bogotá, D. C., Colombia Universidad Nacional de Colombia Universidad Nacional de Colombia Bogotá, D. C. Colombia; 2 Grupo de Investigación Violencia y Salud, Universidad Nacional de Colombia, Bogotá, D. C., Colombia Universidad Nacional de Colombia Universidad Nacional de Colombia Bogotá, D. C. Colombia; 3 School of Public Health, Physiotherapy and Sports Science, University College Dublin, Dublin, Ireland University College Dublin University College Dublin Dublin Ireland; 4 Grupo de Equidad en Salud, Facultad de Medicina, Universidad Nacional de Colombia, Bogotá, D. C., Colombia Universidad Nacional de Colombia Universidad Nacional de Colombia Bogotá, D. C. Colombia; 5 Departamento de Salud Pública, Facultad de Medicina, Universidad Nacional de Colombia, Bogotá, D. C., Colombia Universidad Nacional de Colombia Universidad Nacional de Colombia Bogotá, D. C. Colombia; 6 Faculty of Humanities and Social Sciences, University of Bath, Bath, England University of Bath University of Bath Bath England

**Keywords:** mental health, armed conflicts, violence, warfare, focus group, human migration, salud mental, conflictos armados, violencia, guerra, grupos focales, migración humana

## Abstract

**Introduction.:**

In the Colombian context, research on victims of armed conflict has demonstrated that exposure to violence impacts different aspects of their lives and represents a challenge for their support workers.

**Objective.:**

To explore perceptions, beliefs, and knowledge about mental health and support sources among victims of forced migration -due to the internal armed conflict- and their support workers in Soacha, Colombia.

**Materials and methods.:**

We conducted a qualitative exploratory study. Data were collected from December 2018 to March 2019 using separate focus groups of victims and workers. Thematic content analysis established five deductive categories: perceptions of mental health; the impact of forced displacement and its relationship with mental health; knowledge or perception of institutional support; community networks; and knowledge or perception of the state measures concerning care, assistance, and reparation. We also included some inductive categories that emerged from the analysis.

**Results.:**

Victims demonstrated deteriorated mental health, expressed by emotional, cognitive, and behavioural disturbances. Many of these conducts were exacerbated by the traumatic stress of displacement and exposure to other forms of violence, in addition to social and material deprivation. Emotional avoidance and active search for self-improvement emerged as coping mechanisms used by the victims. Workers experienced high levels of stress assisting trauma victims, and they also needed support for their mental health.

**Conclusions.:**

The findings showed complex and mainly negative impacts on mental health in both groups. Interventions should aim to address poor mental health and strengthen cultural identity and support networks for victims.

## Resumen

**Introducción.:**

Las investigaciones demuestran el impacto del conflicto armado colombiano en diferentes ámbitos vitales de las víctimas, lo que representa un desafío para los trabajadores de apoyo.

**Objetivo.:**

Explorar percepciones, creencias y conocimientos sobre la salud mental y las fuentes de apoyo de las víctimas del desplazamiento forzado por el conflicto armado y sus trabajadores de apoyo en Soacha, Colombia.

**Materiales y métodos.:**

Se trata de un estudio cualitativo y exploratorio. Se recolectó información entre diciembre del 2018 y marzo del 2019, mediante grupos focales de víctimas y trabajadores de apoyo. Los datos recolectados se sometieron a un análisis temático de contenido. Se emplearon cinco categorías temáticas deductivas: percepciones de salud mental, impacto del desplazamiento forzado y su relación con la salud mental, conocimiento o percepción del apoyo institucional, redes comunitarias y conocimiento o percepción de las medidas del Estado concernientes a cuidados, asistencia y reparación. Igualmente se utilizaron categorías inductivas que emergieron durante el análisis.

**Resultados.:**

Las víctimas evidenciaron una salud mental deteriorada, con alteraciones emocionales, cognitivas y conductuales. Muchos de estos comportamientos fueron potenciados por el estrés traumático del desplazamiento y la exposición a otras violencias, además de privación social y material. Como estilos de afrontamiento emergentes, las víctimas emplearon evitación emocional y búsqueda activa de mejoría. Los trabajadores de apoyo describieron su labor como altamente estresante y expresaron la necesidad de recibir apoyo en salud mental.

**Conclusiones.:**

Los hallazgos demuestran un impacto complejo y predominantemente negativo del conflicto armado en ambos grupos. Las intervenciones deben apuntar a abordar los problemas de salud mental en trabajadores y víctimas, y a fortalecer la identidad cultural y las redes de apoyo de las víctimas del desplazamiento por el conflicto armado interno.

Internationally, 51 million people have experienced forced migration ^1^, impacting multiple areas of their lives and communities. These areas include living with different cultures, lifestyles, economies, legal structures, and unstable access to health services. Consecuently, it increases the risk of mental health disorders in the displaced individuals ^2^. People displaced due to armed conflict must relocate to new environments with new customs, behaviour patterns, rules of coexistence, and living norms of new communities ^3^.

In Colombia, Gómez-Restrepo *et al*. ^4^ described mental health disorders and other challenges prevalent in the adult population according to municipalities’ characteristics and violent history. They classified municipalities as those with ongoing (21.8%), discontinued (65.5%), and attenuated (12.7%) conflict and found that the prevalence of anxiety, depression, possible post-traumatic stress disorder, and tobacco smoking was higher in municipalities with ongoing violence.

Access to appropriate resources and tools required to help individuals manage psychological and behavioural changes due to armed conflict influences how well people adapt to and integrate into new social structures. International organizations and societies must recognize the turmoil, destruction, and losses inherent to forced displacement due to armed conflict ^4^^-^^6^ and the long-term -often permanent- mental health effects of these exposures. Toward that aim, interventions, services, policies, and programs to treat and prevent the mental health problems are paramount for assisting traumatized populations, who are often marginalized ^7^. Community-based strategies such as community networks are primary care components in delivering mental health services, promoting integrative health, encouraging family participation in the programs, and fostering the formation of self-help groups ^8^.

In Colombia, research demonstrates that exposure to armed conflict can negatively affect individuals in multiple dimensions. This perspective is key for understanding how individuals react to such conflict ^9^^,^^10^. Community-based research should aim to connect and articulate these social problems using a multidisciplinary qualitative approach ^9^^,^^10^. Qualitative research to explore the effect of armed conflict on the mental health of forced displacement victims allows for co-constructing opinions and perceptions from individuals with shared experiences. It also enables exploring their experiences in the context of broader health determinants, as Dahlgren and Whitehead described ^11^. Forced migration can have negative consequences not only for direct victims but also for the support workers who assist them. Understanding the perceptions of these support workers about the effects of armed conflict on the victims’ mental health is important for co-constructing effective intervention strategies. Therefore, this study aimed to explore the perceptions, beliefs, and knowledge about mental health and sources of support among victims of forced migration -due to the internal armed conflict- and their support workers in Soacha, Colombia.

## Materials and methods

For this qualitative exploratory study, we used two focus groups comprising victims of the armed conflict and their support workers. Data was collected from December 2018 to March 2019. The theoretical framework was underpinned by a thematic content analysis ^12^. Qualitative data collection and analysis techniques were used within a positive paradigm. Participants’ discourses were taken at face value and analysed realistically, without interpretations, using semantic analysis.

### Context of study

The autonomous municipality of Soacha is in the department of Cundinamarca, Colombia. In 2022, the population of Soacha consisted of 808,288 inhabitants and a population density of 4,322.40 inhabitants/km^2^. This population density is higher than the national (45 inhabitants/km^2^) and departmental (142.67 inhabitants/km^2^) ^13^. Additionally, the population of victims of the armed conflict in Soacha has increased since 1985, as the municipality has been recognized as a place to settle by these internal migrants. Soacha is near Bogotá, the capital of Colombia, and provides opportunities for paid employment and access to health and social care facilities and resources. Between 1985 and 2017, 37.15% of the displaced population in Cundinamarca was in the municipality of Soacha ^13^.

*La Unidad para la Atención y Reparación Integral a las Víctimas* (UARIV) is a public national institution that supports integration and reparation for victims of the armed conflict. UARIV’s work also brings the state closer to the victims, offering them an effective, sustainable, and appropriate future.

### Participants and recruitment

Participants for this study were selected with the help of UARIV’s leader. We selected two separate groups:

- A convenience sample of victims of the armed conflict. We approached face to face while they waited to receive information from support workers at UARIV on a weekday morning. Of the 25 individuals who met the inclusion criteria, 16 agreed to participate, and 9 refused. The inclusion criteria were older than 18 years, residing in Soacha, and having been displaced due to the internal armed conflict. Displacement for any other reason was an exclusion criterion.
- A convenience sample of support workers was identified from the UARIV mailings. Thirty individuals met the inclusion criteria, and 13 agreed to participate. The inclusion criteria were being a UARIV worker and being involved in any support services for victims of the armed conflict. The only exclusion criterion was a refusal to participate in the study. In both samples, the reasons for not participating were not explored.

## Data collection

The two researchers developed interview guides prior to conducting the focus groups: a female physician, child psychiatrist, and professor at the *Universidad Nacional de Colombia*; and a female physician, psychiatrist, Ph. D. in public health, and professor at the *Universidad Nacional de Colombia*. Both have research experience in the fields of violence and health and were assisted by a female epidemiologist with background in health and conflict research.

The interview guides covered the five thematic categories described in table 1. These categories were used to classify concepts that shared common characteristics or were related to each other. Categories were divided into two types: deductive, which were created by the researchers at the outset based on their theoretical knowledge of the subject; and inductive, which emerged directly from the collected data and were reviewed without prior consideration, regardless of whether they were new to the theoretical approaches or aligned with them.

**Table 1:** Topic guide for the focus groups and categories for thematic content analysis

Topic guide for the focus groups		Thematic content analysis			
Victims of armed conflict	Support workers who provide care to victims of armed conflict	Categories	Subcategories	Victims of armed conflict	Support workers
1. Self-perceptions of mental health	1. Self-perceptions of mental health	1. Perceptions of mental health	Self-perceptions of mental health	✓	✓
2. Consequences of forced displacement	2. Perceptions of the relationship between mental health and forced displacement		Perceptions of victims’ mental health	✓	✓
3. Perceptions of institutional support	3. Knowledge about institutional care and assistance routes as a care service provider		Services aimed at supporting mental health	✓	✓
			Coping mechanisms of the support workers (inductive)	-	✓
4. Importance of community	4. Community network		Concept of mental health (inductive)	✓	✓
5. Comprehensive care, assistance, and reparation measures	5. Perceptions of the measures offered by the state, related to care, assistance, and reparation.	2. Impact of forced displacement and its relationship with mental health	Problems generated by forced displacement	✓	✓
			Problems with the victims’ mental health generated by forced displacement	✓	✓
			Style of coping with the problems generated by forced displacement	✓	✓
		3. Knowledge and perception of institutional support	Population’s knowledge regarding the services to which they can get access	✓	✓
			Perception of their work: meaning, utility, needs, and improvements	-	✓
			Knowledge of governmental and non-governmental entities providing services to the victims of armed conflict	-	✓
			Barriers to access services (inductive)	-	✓
		4. Importance of community network	Perception regarding the construction of an intersectoral network	✓	✓
		5. Knowledge and perception of the measures offered by the state related to care, assistance, and reparation.	Knowledge of Colombian law	✓	✓
			Support workers’ perception regarding the aid received from the State by the support unit and to victims of armed conflict	-	✓
			Support workers' perception of the country's work in repairing victims of armed conflict	-	✓

Subcategories were defined as elements related within them that collectively formed a category. Themes were defined as category sets that created patterns with shared meanings around a central concept. Subthemes were understood as the elements that together constitute a theme ^14^. In addition, a short questionnaire was designed to collect participants’ sociodemographic characteristics.

Data were collected at the UARIV facilities. All support workers were working in Soacha. Two field research assistants (psychologists) guided the focus groups. Another psychologist compiled the audio recordings and written observations from the focus groups. Before the group discussions began, the field researchers established relationships with the participants by sharing their credentials and explaining the study objectives. It took one session of 90 minutes per group. Only the field researchers and participants were present in the two sessions. Each thematic category was discussed until data saturation was achieved. The results were socialized to the participants in a private group presentation for feedback.

### Analysis

An occupational therapist and a medical student transcribed the audio recordings of the focus groups. Transcripts of the data were not returned to the participants. The researchers developed a coding matrix for each focus group’s interview guide, collated in Microsoft Excel. The authors performed the data coding and the thematic content analysis to identify relevant categories. A hybrid analysis was used for both groups (victims of armed conflict and support workers), based on five deductive categories with their corresponding deductive and inductive subcategories (Table 1).

### Trustworthiness criteria

The following criteria were considered to ensure the accuracy of the research ^14^^,^^15^:

*Credibility*, by presenting the study results to the victims of the armed conflict and support workers so they could check the accuracy of our interpretations from the focus group discussions.

*Dependability*, by documenting and recording all collected data.

*Transferability*, by understanding similar realities presented in care settings for victims with characteristics comparable to those of the participants of this study.

### Ethical considerations

All participants signed an informed consent form before participating in the study. The researchers informed the victims of the armed conflict that the study was independent of the UARIV’s services and explained to the eligibles that their participation was voluntary.

Since the researchers had no connection to UARIV, they reassured participants that their answers or discussions from the focus groups would not affect UARIV’s processes. Authors maintained data confidentiality throughout the study.

The participants were free to withdraw from the study at any time.

The results from the focus groups were presented to the included population in a private group session for feedback. However, this feedback was not included in the analysis.

This study was developed within a larger project on a community-based intersectoral network for managing possible mental health disorders and other problems associated with forced displacement -due to the internal armed conflict- in Soacha, Cundinamarca, Colombia.

The research protocol was approved by the Ethics Committee of the *Universidad Nacional de Colombia*, Bogotá, Acta No. 009-109-19 of 2019.

## Results

Table 2 shows the characteristics of the victims of the armed conflict from the following departments (territorial units): Tolima, Nariño, Huila, Chocó, Caquetá, Sucre, Guaviare, Cauca, and Cundinamarca.

**Table 2:** Characteristics of participants by focus group

Demographic characteristics		Armed conflict victims	Support workers
		(N = 16)	(N = 13)
Age, median (25-75 percentile)		36.5 (33.5-55)	40 (30-46)
Sex, n			
	Women	13	9
	Men	3	4
Marital status, n			
	Single	8	6
	Divorced	1	1
	Widower/widow	2	-
	Married/couple	5	6
Education level, n			
	Middle school	5	-
	High school	9	3
	Technical	2	2
	Bachelor’s degree	-	7
	Postgraduate	-	1
Children, n			
	No	2	3
	Yes	14	10
Occupation, n			
	Housewife	7	*
	Housekeeper	2	
	Kitchen worker	1	
	Hairdresser	1	
	Salesperson	1	
	Freelancer	1	
	Unemployed	2	
	Not responded	1	
Profession, n			
	Administration	*	5
	Psychology		2
	Teaching		2
	Forensic investigation		1
	Social work		2
	Law		1
City of residence, n			
	Bogotá	*	3
	Soacha		8
	Others		2
Years of experience in attention			
to victims (years), n (%)			
	≤2	*	7 (53.8)
	2-6		2 (15.3)
	6-10		2
	≥10		2

* Questions were asked only in one of the focus groups

### Perceptions of mental health

#### Self-perceptions of mental health

Among the victims of armed conflict, the self-perceptions of mental health were mainly related to negative emotions, such as revenge, connected with the violent experiences around forced displacement:

*“In my case, revenge suddenly comes, I have lived things better not to remember” (female victim).*

Resentment towards institutions or entities, such as the government, the armed forces, or the health care system, was reported:

*“Even if they give you an apartment, that does not mean they return to you the lands you lost, the freedom you lost, because, in the countryside, you are free to go wherever you want. Of course, you must be afraid of the guerrilla. Yes! When the armed forces came, you had to hide, but right now, our mindset will never change. We will continue with this pain, even if we consult the psychologist” (female victim).*

The victims of the armed conflict reported symptoms related to exposure to diverse traumatic events in the context of forced displacement, like persistent re-experiencing:

*“You remember the same history over and over again, and live it again, more vividly than when it happened” (female victim).*

Increased anxiety:

*“You hear a hard noise of a car in the street, and you get scared immediately. You experience it like a psychosis, as if you may always be killed” (male victim); numbing and avoidance: “I go out to distract myself and not to think about anything... I say, psychologically, I no longer have a heart, I do not feel, I do not have feelings” (female victim).*

On the other hand, support workers associated their mental health status mainly with emotional distress and burdens generated by their work activities:

*“It is not only the health of the victims, one who is working with them is also severely affected. I, for example, have worked with this vulnerable population for 15 years and worked in the social sector for 30 years, and believe me, nowadays and every day, I listen stories in my work and carry on, I do my work, but I feel helpless and get angry” (male support worker).*

Moreover, support workers reported feeling fatigued assisting people who are often in a bad mood and have a high emotional load:

*“It has happened to me that I have arrived home exhausted, not only mentally but also physically, due to the energy of a person” (male support worker).*

Reports about brooding on some specific facts commented by the victims of the armed conflict:

*“It was very hard because they killed her daughter of nine months, it was the military, so since that woman told me that terrible story, I have it on my head all the time, and all I can do is telling her: let me ask the lawyer” (female support worker).*

#### Perceptions of the mental health of victims of the armed conflict.

Participants in the support workers’ group perceived a mental health affectation and deterioration in victims of the armed conflict that have led to the development of mental health disorders and behavioural problems:

*“I am one of the newest in this job. I come as a representative of the governmental health sector and, in the few days that I have been working here, I have noticed that people looking for assistance have serious problems: drug addiction, suicide attempts, and schizophrenia, especially very young people, a population you would not expect to be so affected by these diseases. It is a wake-up call to realize that this population is vulnerable to those diseases and circumstances, leading them to make that kind of decisions” (female support worker).*

The support workers highlighted an implicit request by these victims to be heard and accompanied in the search for their well-being.

#### Perceptions of provided services aimed at supporting mental health

Some of the victims of the armed conflict reported that they had received psychological interventions for their mental health symptoms and had experienced a transitory relief:

*“I am not saying that they did not help me. I consulted many psychologists who helped me a little, but how can I explain it? They give you comfort, but then, when you are alone, it is worse: you come back, remember it, and ask yourself why, why?” (female victim).*

Expressions of dislike and refusal of psychological interventions were frequent:

*“This is like you have a healing wound, and they come back with a stick to poke at it again and again” (male victim).*

These victims also perceived that the healthcare system institutions were not providing adequate support, or the services required to improve their mental health:

*“Mental health exists in words, but in reality, in practice, it does not exist in Colombia. For those of us who have lived through armed conflict, for those who have had our family killed, what kind of mental health -or health in general- can we have? How do you think we feel?” (male victim).*

In the group of support workers, the participants reported that some of the victims probably had avoided getting in contact with services caring for mental health due to the emotional consequences of their involvement in the armed conflict:

*“All [victims of the armed conflict] need humanitarian aid, but mental health assistance is also very important. Just a few of them receive it... Many recognized that they needed this assistance even for events that happened to them 15 years ago. Some may have never shared with their families what happened because they could feel ashamed. So not only victims but also ex-combatants -those people who have suffered due to very tough situations- also need assistance” (make support worker).*

From the support workers’ perspective, even if they are qualified for their job, providers of mental health care face important challenges in the assistance of victims due to the armed conflict:

*“When you are a psychologist, you studied for that [confront complicated situations], you are supposed to handle it, but I think it is not necessarily true. I assisted up to three victims of sexual abuse in one day, and that is really hard to do. Trying to be cold and not to worry. When I began to work with armed conflict victims, my first week was very difficult because when you assist a victim of torture, it really touches you” (male support worker).*

Regarding the services available to care for their own mental health, participants in the support workers’ group emphasized the need to be heard:

*“It is important; we are always there for the others, but who listens to us?” (female support worker). They also requested mental health-promoting activities, spaces to relax and share their work experiences: “It would be invaluable for us to have a space, for example, where we could meet at least once a month and talk about how we feel about the cases we have had and the situations we have faced” (female support worker).*

#### Coping mechanisms of the support workers (inductive)

This subcategory emerged in the support workers’ group. They reported differentiating contexts by separating family and work environments. They highlighted the need for a space where they could discharge their emotions, leaving aside worries and tensions that could hinder the attention to the victims of the armed conflict: “I worked for many years with dead people and learned that work is work and home is home. I have a family -my wife, and my son-, I forget all when I am at home. I love my family, and everything else ends there” (male support worker).

#### Concept of mental health (inductive)

Four approaches to mental health emerged from the thematic analysis of the previous subcategories: mental health as a state of well-being, as an emotional state, as a disease, and as a process influenced by emotional expression and interaction with others. The latter was particularly prevalent in participants’ testimonies from both groups, specifically around the allusion to improvements in emotional distress that some perceived when interacting with each other or with external agents who provided spaces for relaxing and sharing common experiences.

### Impact of forced displacement and its relationship with mental health

#### Problems generated by forced displacement

Victims of the armed conflict reported many difficulties coping with the urban living conditions and having been rejected and stigmatized when people of their new community identified them as victims of the armed conflict coming from rural areas:

*“It was a drastic change, coming from a place where you are your own boss, and then going where nobody knows you and searching for a job when you do not know how to take a bus. The first time I took a bus, I fell down... When you are searching for a job, you have to be honest: when I say I came from a farm, people tell me that I do not know how to do anything; when they ask me where I come from, and I answer I am displaced, they think I am part of the guerrilla” (male victim).*

They pointed out their disadvantaged economic status and loss of family and social support networks resulting from the forced displacement. Furthermore, a loss of identity was evident when assuming a new role in the community, characterized by perceptions of vulnerability. The negative economic situation compounded their other negative feelings. These included revenge, frustration, sadness, loneliness, helplessness, pain, rage, anxiety, guilt, insecurity, mistrust, uncertainty, anguish, fear, and isolation.

The support workers reported the difficulty of the victims adapting to the new cultural and social conditions in the place of destination since they had faced a high number of stressors, including difficulty in accessing employment and health services.

#### Mental health problems of the victims of the armed conflict generated by forced displacement

Participants in the two focus groups stated that the difficulties of the victims in coping with the social and cultural stressors and the lack of education and employment opportunities had contributed to the deterioration of their mental health. Support workers linked overcrowding or an increase in the number of people arriving at Soacha in displacement conditions to difficulties of the victims of the armed conflict in accessing resources and opportunities. However, participants in the two focus groups reported that the mental health problems of the victims were mainly related to the traumatic events that surrounded forced displacement, such as sexual violence, torture, having witnessed homicides, and other types of violence. The support workers emphasized that some victims of the armed conflict held resentment throughout their lives, accentuated by not receiving timely and appropriate psychological attention and other support services.

#### Coping mechanisms of the victims of the armed conflict with the problems generated by forced displacement (inductive)

This subcategory emerged from the thematic analysis and concerns about the two coping mechanisms identified in victims of the armed conflict to handle adverse situations associated with their forced displacement: coping/avoidance (to address their victimization and to seek psychosocial support for any problems that they may be experiencing); and search for self-improvement (attempts made by victims to enter the workplace and school, both providing continuity to their lives without focusing solely on their experiences of victimization):

*“I know we will never recover everything we lost but we must help ourselves. If we come here and do not get a house... We are not going to cry forever; we have to move on... The social organization I joined here in Soacha supported me. Then, I set up my business and began to sell coffee, and I still have my project. I belonged to another organization where I took training courses and learned to make espadrilles. My son had learned to make handicrafts. We both have managed to make it through” (female victim).*

The second coping strategy, related to an active search for selfimprovement, aligns with the ‘emotional barriers’ that victims of armed conflict reported as a means of self-protection against being emotionally affected again:

*“I arrived with nothing, and little by little, I have built my life. Now, I am an assistant, and I want to pursue a technician degree because I understand that it is the only way to provide my family with a better life. By crying and regretting, I will never get anything” (female victim).*

### Knowledge and perception of institutional support

#### Population’s knowledge regarding the services they can access.

Besides negative experiences with mental health care services, some victims of the armed conflict reported difficulties in requesting information about other services and how to access them, such as economic support and educational and job opportunities. They felt that support workers were not aware of all the services that could be available to help and support them and also perceived the support workers as hostile, aggressive, defensive, and not empathetic. This attitude made them feel misunderstood and frustrated:

*“It is very sad because they do not give you the support you need. They just do not. You come here and ask them a question about something and. Nothing. They tell you it [the required service] has to do with another service provider; it has to be around here and around there; you have to wait for a turn; they just do not know where and whom you could consult.” (female victim).*

However, the support workers reported that victims of the armed conflict often had unreasonable expectations due to a generalized lack of information concerning the different support from institutions and services available to them:

*“They really do not know anything about procedures to access health services; they have no idea of how to get a job. I think they do not know any of the routes” (male support worker).*

#### Perception of the work

This subcategory was identified in the support workers’ group, where participants mentioned different stressors in their work impacting the effective advice they could provide, such as exposure to stories of extreme violence experienced by some of the victims of the armed conflict. The support workers felt they needed training to help them deal with these circumstances:

*“The point is that we should be trained in how to talk with people because in my case, yesterday a woman came (I am not a psychologist), and I really did not know what to tell her. I only told her that she needed psychosocial therapy” (female support worker).*

Moreover, support workers reported that an unstable internet connection affected their work and concentration when trying to listen and pay attention to the stories narrated by the victims of the armed conflict.

Despite perceiving their working conditions as highly stressful, some support workers described the positive aspects they offered to the victims, such as active listening, empathy, tolerance, and patience. They felt that their work was gratifying and valuable in providing a safe place where the victims of the armed conflict could be heard.

#### Knowledge of governmental and non-governmental entities providing services to victims of the armed conflict

This subcategory (identified in the support workers’ group) alludes to the importance of being informed about other entities and institutions providing support to victims of the armed conflict and how to access the services. The support workers considered that the assistance obtained from other institutions contributes to creating a communication channel when a certain service is required by the victims of the armed conflict. However, they also reported insufficient knowledge about programs and services provided by other entities that work with this vulnerable population.

#### Barriers to access services (inductive)

This subcategory emerged in the support workers’ group. Regarding access to health and mental health care services, the support workers reported obstacles imposed by the entities in charge of the health coverage for the general population in Colombia:

*“Unfortunately, here in Soacha, there are health-promoting companies [EPS] that do not guarantee the coverage of the services demanded or needed by them [the victims of armed conflict], I think this problem might be audited or negotiated with those entities to ensure the provision of the services required, regardless of where this population resides, because they are settled in different places, and therefore, accessing those services is more complex for them” (female support worker).*

Concerning education services, the support workers mentioned barriers related to a lack of schools in the municipality of Soacha coupled with a temporary interruption of humanitarian aid.

### Community network

#### Perception regarding the construction of an intersectoral network to support the victims of the armed conflict

When enquiring about the importance of community in their lives, some victims recognized that belonging to a community was like belonging to a family. They stated that a sense of community enabled them to have close relationships with others, which helped alleviate feelings of loneliness and made them feel capable of achieving goals. Some pointed out that their new communities were characterized by closeness to others and trust, allowing them to advance in handling the emotional impact of their forced migration and exposure to armed conflict. However, they immediately conceded that their forced displacement affected previous community experiences:

*“When you talk about a community, this is like Guaque town, is not it? That is a community! Everyone knows everyone, everyone talks with each other and helps each other when someone needs help, but here, if you do not have food, you do not eat; nobody lends you money like in your town, not here. This [group] is not a community. Here, everyone lives individually. No one cares if you starve” (female victim). Some victims of armed conflict did not feel part of any community; instead, they perceived themselves as “strange creatures that individuals look at” (female victim).*

On the other hand, the support workers mentioned a lack of follow-up of the programs and services of care and support provided by other institutions to the victims of the armed conflict, pointing out the need to have an optimal intersectoral network that allows collaborative work with this population to generate higher effectiveness when addressing solutions to their needs.

### Knowledge and perception of the measures provided by the state, related to care, assistance, and reparation of victims of the armed conflict

#### Knowledge about Colombian law

In Colombia, “The Victims and Land Restitution Law” (Ley 1448 del 2011) determines care, assistance, and comprehensive reparations for victims of the internal armed conflict. In the group of support workers, participants stated that victims had an erroneous knowledge of the law:

*“People [victims of the armed conflict] come here saying that we have to give them a house because of the law; they keep saying we have to give them some money, so we have to stop them. They come with the idea that we will give them lots of money, but obviously this [idea] has nothing to do with what they have suffered” (female support worker).*

The group of victims of the armed conflict identified the need to better understand the purpose of the law. They stated they only had access to the specific information they asked for. Instead, they needed a comprehensive overview of their full entitlement under this law:

*“The documents they give you contain a lot [of information] about the law, but you really do not understand what the law actually includes or how it applies to us” (female victim).*

Support workers’ perception regarding the aid provided by the state to the support unit -UARIV- and the victims of the armed conflict

The support workers identified that victims of the armed conflict were perceived by the state of Colombia as ‘a problem’, placing the responsibility for their care on the support workers themselves. Consequently, support workers felt abandoned by the state:

*“One feels abandoned by this unit, maybe because it is part of the state and receives too many aids, has too much budget. We could be spoiled ones, with a better salary and activities because working with people is my passion. I like it, but believe me, nobody considers our mental and physical state. We are totally neglected, and this is really sad” (female support worker).*

Likewise, they perceived deficiencies regarding the services and resources provided by the country to the victims of the armed conflict. On some occasions, the support workers used their own resources trying to solve problems they would expect the state to cover:

*“Sometimes I have to use my own mobile and internet to call to a health entity and ask for help, maybe to solve a problem. This is not a true solution because the expectations are huge, and it is little what you have to give” (male support worker).*

Support workers’ perception of the country’s measures in repairing victims of the armed conflict

Support workers referred to the way the country perceives the victims of the armed conflict:

*“Victims are a problem, a burden” (female support worker).*

Due to the inadequate coverage of the victims of the armed conflict and their needs, support workers believed this population has not been a priority for the state. They also considered that compliance with the law had been only partial regarding the mistreatment received by the victims. Additionally, the support workers indicated that the change of government every four years had strongly influenced the provided care service and the designated resources to the victims, as each new administration prioritized different sectors according to its agenda.

### Mental health as a relational fact and its implications for the care and assistance of victims of the armed conflict and support workers

A higher-order category for mental health emerges from the relationships among individuals and between individuals and their social environments, facilitating the formation of emotional bonds. For both groups, mental health is associated with a collective identity, the opportunities afforded by participation in protective social networks, and access to resources that ensure a ‘dignified life’ in resettlement areas. Consequently, both groups concur that mental health interventions should extend beyond individual psychological approaches.

## Discussion

In this study, the mental health of the victims of the armed conflict was linked to their awareness of their own decisions and actions, their emotional suffering from long-lasting traumatic memories, and their new disadvantaged socioeconomic conditions. For the support workers, their mental health reflected emotional and physical distress related to caring for the extensive needs of the victims of forced internal displacement due to the armed conflict. These results contrast with previous research involving also support workers of victims of the armed conflict in Colombia, whose mental health was characterized by physical, psychological, and social well-being (including social relationships) ^16^. Similarly, Cardona-Duque *et al*., who used focus groups of community members to explore the relationship between mental health and political violence, identified mental health as a state that includes feelings of tranquillity, trust, and acceptance ^17^.

Regarding the victims of the armed conflict, now living in Soacha, the negative emotions resulting from their exposure to armed conflict were significant indicators of their impaired mental health. However, qualitative research with victims of armed conflict found that good mental health was possible if the victims could overcome negative emotions and find coping strategies to rebuild their lives ^18^. Some people affected by violence report that their mental health depends on the harmony and balance between physical, social, psychological, spiritual, and emotional well-being ^19^^,^^20^, which is only achievable when basic needs are met ^21^. This finding concurs with Maslow’s hierarchy of needs, at the top of which is self-actualization -‘being fulfilled and happy’- attainable only after addressing other levels, such as physiological, safety, feeling loved or belonging, and having positive self-esteem ^22^.

Concerning mental health and forced displacement, participants in both focus groups recognized the negative impacts on victims of the displacement itself and those witnessing the horrors of the armed conflict. Those effects include long-lasting emotional pain, resentment, revenge, distrust, and other manifestations of traumatic stress. The victims of the armed conflict reported -and the support workers observed- challenges in adapting to their new cultural frameworks and worsened economic situations after resettlement. Our findings of low satisfaction with the available support and health care services concurred with other studies ^23^^,^^24^. In addition, the traumatic re-experiencing when accessing services and perceptions of poor empathy - from support workers and health professionals- were similar to recent results for service provision to victims of the armed conflict in Meta, Colombia ^25^.

Contrary to the perceptions of the victims resettled in Soacha, the support workers described empathy, active listening, patience, and tolerance as positive aspects of their work with this population. They added that barriers in health services impeded victims of the armed conflict from processing their traumatic experiences, leading, in some cases, to the emergence of mental health disorders, suicide attempts, and risky behaviours, such as psychoactive substance use. Castaño *et al*. identified that the higher prevalence of mental health disorders and drug use among victims of the armed conflict in Colombia, compared to the general population, justifies a service focus on mental health, recovery, and wellness ^26^. Although institutional programs exist to promote wellness and provide specialized mental health services, the support workers of Soacha added to be unaware of the procedures for referring victims of the armed conflict to them ^27^.

In this study, the victims of the armed conflict and support workers recognized the importance of resilience. Victims of the armed conflict could use this attribute to move on and overcome the negative consequences of forced displacement, consistent with coping strategies previously described by Di-Colloredo *et al*. regarding displaced persons in Colombia ^28^. Mental health interventions for this group should focus on helping victims develop emotional resilience to overcome the effects of their exposure to the armed conflict and their subsequent displacement. The literature reports that using support workers to deliver mental health interventions can help victims of the armed conflict overcome feelings of anguish, hatred, sadness, and suffering resulting from their experiences in the armed conflict. These interventions enable the victims to deploy positive adaptation mechanisms, aiding in emotional recovery and appropriate problem resolution. ^16^^,^^21^^,^^29^.

Furthermore, support workers reported an emotional burden stemming from the demanding attitudes of victims, based on insufficient information and unrealistic expectations about the services provided. This finding supports earlier work demonstrating that stressors linked to the care of victims affect the care provision process ^27^. Moreover, other studies involving personnel working with victims of the armed conflict found that support workers were dissatisfied with the mental health support they received ^16^^,^^29^. Support workers often felt that the government had abandoned them and was unsupportive towards them in their efforts to assist the victims of the armed conflict ^16^^,^^30^. This perception of abandonment, shared by the victims and the support workers, contradicts the country’s establishment of UARIVs as a comprehensive reparation mechanism.

One notable observation was that the support workers interviewed in Soacha described experiencing ‘empathy-based’ stress from vicariously witnessing the victims of the armed conflict’ traumas, which led to strain and adverse health reactions, among other effects. A consistent relationship exists between the selfperceived mental health of the victims, marked by negative emotions and post- traumatic symptoms, and that of the support workers, who exhibited symptoms of vicarious stress and chronic fatigue. The self-perception of the support workers’ mental health contradicts their expected role of providing support to the victims of the armed conflict and may explain the hostility experienced by these victims in their interactions with the support workers.

Empathy-based stress has also been called compassion fatigue, secondary traumatic stress, and vicarious traumatization ^31^. The support workers observed in this study should receive help to manage the emotional effects of repeated exposure to victims’ trauma stories. Supporting these workers in their care for the welfare of the victims of the armed conflict is crucial to sustaining their efforts. Strategies could include identifying sources of individual and collective stress and detecting early emotional reactions; establishing clear roles and responsibilities and maintaining fluid internal communication in the workplace; ensuring adequate training for team members; and creating spaces for recreation, reflection, and mutual support among coworkers ^16^. Overall, mental health interventions for victims of forced internal displacement resulting from armed conflicts should aim to integrate displaced persons into existing similar communities while understanding that, for many, all they have left is their identity. Reintegration into new communities facilitates rebuilding the social fabric ^20^.

The main strength of this study is exploring the complex phenomenon of mental health problems associated with forced displacement due to the internal armed conflict from the perspectives of two types of social actors: the victims of the armed conflict and the support workers attempting to care for them. The study’s main limitations include the reliance on convenience sampling to recruit participants. Furthermore, the study did not explore any demographic differences that might have explained varied opinions and beliefs among participants.

Qualitative exploratory studies are a research approach used to investigate poorly understood topics or those with limited existing information. This study focused on exploring perceptions, beliefs, and knowledge about mental health, providing a foundation for future research to analyse higher-order topics, such as the diversity of experiences portrayed by the participants. However, the concept of mental health -a phenomenon arising from social relationships- emerged as a high-order theme encompassing the diversity of experiences. This finding should be considered when planning mental health interventions for this population, as it transcends intrapsychic and individual approaches.

It is essential to distinguish between expected reactions to trauma exposure and psychiatric symptoms requiring medical intervention in the studied populations. This distinction is crucial for addressing the mental health needs of both groups. The terms “mental health” and “symptoms” were used according to the theoretical models accepted by the researchers, reflecting their foundational training as medical and health professionals. Consequently, the analysis was grounded in biomedical theoretical foundations. The participants’ self-perceptions (labelled as mental health and symptoms) corresponded to expressions of well-being or discomfort. Future research should code this from the participants’ perspectives.

The findings from the focus groups with victims of the armed conflict and support workers in Soacha revealed complex and predominantly negative effects on their mental health. In the context of an ongoing armed conflict like the Colombian one, the people using the services of UARIV are those feeling safe enough to express openly their status as victims of the armed conflict. They have achieved recognition and inclusion in the Single Registry of Victims. Therefore, we assume that the included population of victims may represent the less vulnerable compared to others, who, despite needing the service, do not use it due to fear of being identified or the inability to achieve official recognition as a victim of the armed conflict.

The victims of the armed conflict in this and similar studies have experienced various traumatic events that, coupled with new socioeconomic precariousness, results in a significant mental health burden. In turn, providing support to these victims of forced internal displacement negatively affects the mental health of the support workers. Notably, many victims of the armed conflict in our focus group felt that their support workers were not very helpful, while the support workers believed they were providing empathetic services. Future researchers could explore possible reasons for this discrepancy in perceptions.

Beyond their suffering, the victims of armed conflict were aware of their capabilities and were resourceful in managing their situations. Meanwhile, the workers experienced high levels of stress and required support for their own mental health due to their experiences of working with armed conflict victims.
